# Transcriptomic insights into the mechanism of action of telomere-related biomarkers in rheumatoid arthritis

**DOI:** 10.3389/fimmu.2025.1585895

**Published:** 2025-05-22

**Authors:** Lijuan Feng, Kaiyong Bai, Limeng He, Hao Wang, Wei Zhang

**Affiliations:** ^1^ Department of Nuclear Medicine, Sichuan Provincial People’s Hospital, University of Electronic Science and Technology of China, Chengdu, Sichuan, China; ^2^ Department of Nuclear Medicine, The First Affiliated Hospital of Kunming Medical University, Kunming, Yunnan, China

**Keywords:** rheumatoid arthritis, autoimmune diseases, telomeres, biomarkers, bioinformatics analysis, *in vitro* experiment

## Abstract

**Background:**

Rheumatoid arthritis (RA) is an autoimmune inflammatory disease. The mechanism by which telomeres are involved in the development of RA remains unclear. This study aimed to investigate the relationship between RA and telomeres.

**Methods:**

In this study, we identified differentially expressed genes (DEGs) between RA and control samples by analyzing transcriptome data from a public database. Candidate genes were determined through the intersection of DEGs and telomere-related genes. Biomarkers were subsequently identified using machine learning algorithms, receiver operating characteristic analysis, and expression level comparisons between RA and control samples. Additionally, a nomogram model was employed to predict the diagnostic ability of biomarkers for RA. Subsequently, the potential mechanisms of these biomarkers in RA were further explored using gene set enrichment analysis (GSEA), subcellular localization, chromosome localization, immune infiltration, functional analysis, molecular regulatory networks, drug prediction, and molecular docking. Furthermore, the expression of biomarkers between RA and control samples was validated through *in vitro* experiments.

**Results:**

ABCC4, S100A8, VAMP2, PIM2, and ISG20 were identified as biomarkers. These biomarkers demonstrated excellent diagnostic ability for RA through a nomogram. Most of the biomarkers were found to be enriched in processes related to allograft rejection and the cell cycle. Subcellular and chromosomal localization analyses indicated that ABCC4 is localized to the plasma membrane, ISG20 to the mitochondria, PIM2 and S100A8 to the cytoplasm, and VAMP2 to the nucleus. Additionally, nine differential immune cells were identified between RA and control samples, with a strong correlation observed between the biomarkers and activated CD4 memory T cells. S100A8, PIM2, and VAMP2 exhibited high similarity to other biomarkers. Furthermore, three transcription factors (TFs), 121 microRNAs (miRNAs), and six long non-coding RNAs (lncRNAs) were identified as targeted biomarkers. Five drugs—methotrexate, adefovir, furosemide, azathioprine, and cefmetazole—were also identified as targeted biomarkers. Notably, ABCC4 interacted with all five drugs and exhibited the strongest binding energy with methotrexate. The results of the *in vitro* experiments were consistent with those obtained from the bioinformatics analysis.

**Conclusion:**

This study identified five biomarkers—ABCC4, S100A8, VAMP2, PIM2, and ISG20—and offered new insights into potential therapeutic strategies for RA.

## 1 Introduction

Rheumatoid arthritis (RA) is a chronic, autoimmune inflammatory disease typically characterized by persistent morning stiffness, joint pain, and swelling (1). Severe cases can lead to destruction of articular cartilage and bone (1). Currently, the global prevalence of RA has risen to 1.3 percent (2). The etiology and pathogenesis of RA are complex (3), involving a series of inflammatory responses in tissues triggered by a combination of genetic, infectious, and environmental factors (4). The diagnosis of RA primarily relies on clinical symptoms and physical signs, as well as laboratory and imaging examinations (5, 6). Consequently, early, atypical, or inactive RA can easily be overlooked (7). The therapeutic approach for RA involves disease-modifying antirheumatic drugs, nonsteroidal anti-inflammatory drugs (NSAIDs), and biologics (8). Although NSAIDs can alleviate pain and morning stiffness associated with RA, reduce inflammation, and prevent disease progression, their efficacy is often limited. Additionally, NSAIDs may pose risks of gastrointestinal and cardiac toxicity (9). Therefore, further exploration of the specific etiology, regulatory molecules, and underlying mechanisms of RA was essential for improving prevention and treatment strategies.

Telomeres are protein-DNA complexes at the ends of linear chromosomes in eukaryotic cells, which protect chromosome ends from illegitimate ligation and resection (10). Telomerase, a ribonucleoprotein enzyme composed of an RNA template and a catalytic protein, counteracts telomere shortening by synthesizing telomeric DNA (11). Leukocyte telomere length in peripheral blood not only reflects telomere length in other tissues but also indicates the senescent state of immune-related cells in the circulating immune system (12, 13). In recent years, the role of telomere length, telomerase, and its associated protein complexes in the pathophysiology of autoimmune diseases has become a research hotspot (14, 15). Multiple studies have demonstrated that telomere shortening is associated with an increased risk of RA (16). Another meta-analysis revealed that telomere length in RA patients is significantly shorter than that in healthy controls. Telomere shortening was often correlated with increased oxidative stress and an exaggerated inflammatory response, both of which were critical factors in the progression of RA (17). Research also indicates that defects in telomerase activity can lead to accelerated telomere attrition in T-cells, triggering immune dysfunction in RA (15). This dysfunction may exacerbate autoimmune responses and increase disease severity. However, the relationship between telomeres and the onset of RA remains incompletely understood. Therefore, further investigation into telomeres was expected to provide new insights into the pathophysiology of RA and offer potential pathways for its treatment.

In this study, we screened and identified telomere-related genes (TRGs) associated with RA using public datasets. Machine learning techniques were employed to identify biomarkers of TRGs in RA, followed by bioinformatics analyses, including Gene Set Enrichment Analysis (GSEA), immune infiltration analysis, and molecular regulatory network analysis. Additionally, we validated the expression differences of these biomarkers between RA and control samples through reverse transcription quantitative PCR (RT-qPCR). This study aims to analyze, screen, and validate the biomarkers of TRGs associated with RA to enhance the understanding of the pathophysiology of RA and provide evidence for the development of new diagnostic or drug treatment targets.

## 2 Materials and methods

### 2.1 Data collection

The transcriptome data of RA (GSE89408 and GSE55235) were downloaded from the Gene Expression Omnibus (GEO) database (https://www.ncbi.nlm.nih.gov/geo/). GSE89408 (GPL11154) was the training set, including 150 RA tissue samples and 28 control tissue samples; the remaining samples need to be eliminated. GSE55235 (GPL96) was the validation set, including 10 RA tissue samples and 10 control tissue samples; the remaining samples also need to be eliminated. The 2,086 TRGs were downloaded from the TelNet database (http://www.cancertelsys.org/telnet/) (18) (**Supplementary Table 1**).

### 2.2 Identification of differentially expressed genes

The DEGs between RA and control samples in GSE89408 were obtained via the “DESeq2” package (v 1.42.0) (19) (RA vs. control;adj.*P* < 0.05, |log_2_FC| > 1). The DEGs were displayed by volcano plot and heat plot. According to the log_2_FC value, the volcano plot displayed DEGs via the “ggplot2” package (v 3.5.1) (20), with the top 10 up/down-regulated genes marked, and the heat plot displayed DEGs between RA and control samples via the “pheatmap” package (v 1.0.12) (21).

### 2.3 Identification and function of candidate genes

The candidate genes were obtained by the intersection of DEGs and TRGs via the “ggvenn” package (v 0.1.10) (22). The GO and KEGG were employed to analyze the pathways and biological functions involved in candidate genes via the “clusterProfiler” package (v 4.10.1) (23) (adj.*P* < 0.05). GO analysis included biological processes (BP), molecular function (MF), and cellular components (CC). According to the *P*-values, which were sorted from smallest to largest, the top 10 pathways of GO analysis and the top 10 pathways of KEGG analysis results were displayed via the “ggplot2” package (v 3.5.1).

### 2.4 Gene interaction at the protein level

The PPI network of candidate genes was constructed to explore candidate gene interactions at the protein level via the STRING database (https://string-db.org/). The confidence degree was greater than 0.7. The result was shown via Cytoscape (v 3.10.2) (24).

### 2.5 Machine learning

The LASSO algorithm and SVM-RFE algorithm were used for further gene screening based on candidate genes, respectively. In GSE89408, the “glmnet” package (v 4.1-8) (25) was performed with the LASSO algorithm to screen candidate genes according to the minimum lambda value. The “e1071” package (v 1.7-14) (26) was also performed by the SVM-RFE algorithm to screen candidate genes based on error rate and accuracy; the genes output at the lowest error rate were used for subsequent analysis. The genes obtained by the 2 algorithms were intersected to obtain core genes via the “ggvenn” package (v 0.1.10).

### 2.6 Receiver Operating Characteristic (ROC) analysis and gene expression level verification

ROC analysis and expression level verification were employed to obtain biomarkers. In all samples of GSE89408 and GSE55235, ROC analysis was performed to explore the ability of core genes to distinguish RA from control samples via the “pROC” package (v 1.18.5) (27). An AUC value greater than 0.7 indicated that genes had excellent diagnostic ability. The genes passed ROC analysis were candidate biomarkers. In all samples of GSE89408 and GSE55235, expression level verification was performed to explore the expression difference and tendency of candidate biomarkers in RA and control samples. The expression difference between RA and control samples was performed via the Wilcoxon test (*P* < 0.05). Genes with significant differences between RA and control samples, along with and consistent expression trends in both training and validation sets, were regarded as biomarkers.

### 2.7 Construction of nomogram

The nomogram was employed to explore the ability of biomarkers to predict the incidence of RA. In all samples of GSE89408, the nomogram was constructed based on biomarkers via the “rms” package (v 6.8-1) (28). According to the nomogram, biomarkers were pointed separately; each biomarker corresponded to a point, and the points of each biomarker were added together to correspond to the total points. The higher the total points, the higher the risk of RA. The ROC curve was created via the “pROC” package (v 1.18.5), the calibration curve via the “rms” package (v 6.8-1), and the decision curve via the “rmda” package (v 1.6) (29) to evaluate the accuracy of the nomogram. AUC > 0.7, calibration curve slope close to 1, and net benefit > 0 indicated the nomogram was accurate.

### 2.8 Gene Set Enrichment Analysis

GSEA was employed to explore the biological functions of biomarkers for RA. The reference set was “c2.cp.kegg.v7.4.symbols.gmt” based on the Molecular Signatures Database via the “clusterProfiler” package (v 4.10.1) (http://www.gseamsigdb). In all RA and control samples of GSE89408, the Spearman correlation analysis between each biomarker and all the remaining genes was performed via the “psych” package (v 2.4.3) (30). After the correlation coefficients were ranked from greatest to smallest, GSEA was performed, and the first 5 pathways were presented via the “enrichplot” package (v 1.22.0) (31) (*P* < 0.05).

### 2.9 Subcellular and chromosome localization analyses

Subcellular and chromosome localization analyses were employed to explore the distribution of biomarkers in protein subcells and chromosomes. Subcell localization was predicted by the CELLO v.2.5 database (http://cello.life.nctu.edu.tw/). Chromosome localization was performed by the “RCircos” package (v 1.2.2) (32).

### 2.10 Immune infiltration analysis

Immune infiltration analysis was performed to explore the immune cell infiltration in RA. In all samples of GSE89408, the infiltration abundance of 22 immune cells (33) between RA and control samples was performed through the CIBERSORT algorithm and displayed through the “ggplot2” package (v 3.5.1) (*P* < 0.05). To obtain differential immune cells between RA and control samples, the Wilcoxon test was performed, and the result was shown via the “ggplot2” package (v 3.5.1) (*P* < 0.05). Spearman correlation analysis was employed to explore the correlation between biomarkers and differential immune cells via the “psych” package (v 2.4.3) (|correlation (cor)| > 0.3, *P* < 0.05); the result was presented via the “pheatmap” package (v 1.0.12) and the “ggpubr” package (v 0.6.0) (34).

### 2.11 GeneMANIA and Friends analysis

GeneMANIA and Friends analysis were employed to explore the interaction between proteins encoded by biomarkers and other proteins with related roles and functional similarities between biomarkers and other genes. GeneMANIA was performed by the GeneMANIA database (http://www.genemania.org/). Friends analysis was performed by the “GOSemSim” package (v 2.33.0) (35).

### 2.12 Construction of molecular regulatory network

The molecular regulatory network was employed to explore the regulatory relationship between biomarkers and regulatory molecules composed of transcription factors (TFs), miRNAs, and lncRNAs. The TFs-targeted biomarkers were predicted by the TRRUST database (https://www.grnpedia.org/trrust/). The miRNA-targeted biomarkers were predicted by the miRmap database (https://mirmap.ezlab.org/), the DIANA-microT database (http://diana.imis.athena-innovation.gr/DianaTools/index.php), and the TargetScan database (http://www.targetscan.org/). The key miRNAs were obtained by the intersection of 3 databases’ results for subsequent analysis. The lncRNAs based on the key miRNAs were predicted by the miRNet database (https://www.mirnet.ca/), the starBase database (http://starbase.sysu.edu.cn/), and the LncBase v.2 database (https://dianalab.e-ce.uth.gr/html/diana/web/index.php?r=lncbasev2). The TF-mRNA and mRNA-miRNA-lncRNA regulatory networks were presented via Cytoscape (v 3.10.2).

### 2.13 Drug prediction and molecular docking

To obtain potential drugs targeting biomarkers, the DGIdb database (https://www.dgidb.org/), the CTD database (https://ctdbase.org/), and the DSigDB database (http://dsigdb.tanlab.org/) were employed to predict drugs. Drugs shared by the 3 databases were used for subsequent network construction. The drug-biomarker network was presented via Cytoscape (v 3.10.2).

To explore the binding ability between biomarkers and drugs, the molecular docking between the biomarkers and the drugs obtained based on the database was performed. The 3-dimensional molecular structure formula of the drug was downloaded from the PubChem database (https://pubchem.ncbi.nlm.nih.gov/). The protein structure of biomarkers was downloaded from the Protein Data Bank database (http://www.rcsb.org). AutoDock Vina (v 1.2.5) (36) was employed to perform molecular docking. The 3 most powerful combinations were presented by PyMol.

### 2.14 Clinical sample validation

The biomarker expression difference between the RA and control samples was verified by RT-qPCR. A total of 5 pairs of whole blood samples were obtained at Sichuan Provincial People’s Hospital, University of Electronic Science and Technology of China, including 5 RA and 5 controls. The informed consent form needed to be signed and filled out by all participants; approval was obtained from the institutional review board (No. 2023-287). Firstly, the total RNA of 5 pairs of whole blood samples was derived by TRIzol reagent (Ambion, U.S.A.). The RNA concentrations were measured by the NanoPhotometer N50. Secondly, mRNA was reversely transcribed into cDNA utilizing the cDNA Synthesis Kit (Servicebio, Wuhan, China). Finally, **t**he RT-qPCR was conducted. The reagents, conditions, and primers required for the experiment were listed in **Supplementary Table 2**. The expression levels of biomarkers between RA and control samples were calculated by 2^-ΔΔCt^. The internal reference gene was GAPDH, which was employed to normalize the results.

### 2.15 Statistical analysis

Bioinformatics analyses were performed utilizing the R programming language (v 4.3.1). The Wilcoxon test was performed to compare the differences between 2 groups. *P* < 0.05 was considered statistically significant. Expression differences between RA and control samples were measured by t-test in the RT-qPCR experiment (*P* < 0.05).

## 3 Results

### 3.1 Functional analysis of candidate genes

**Figure 1:**
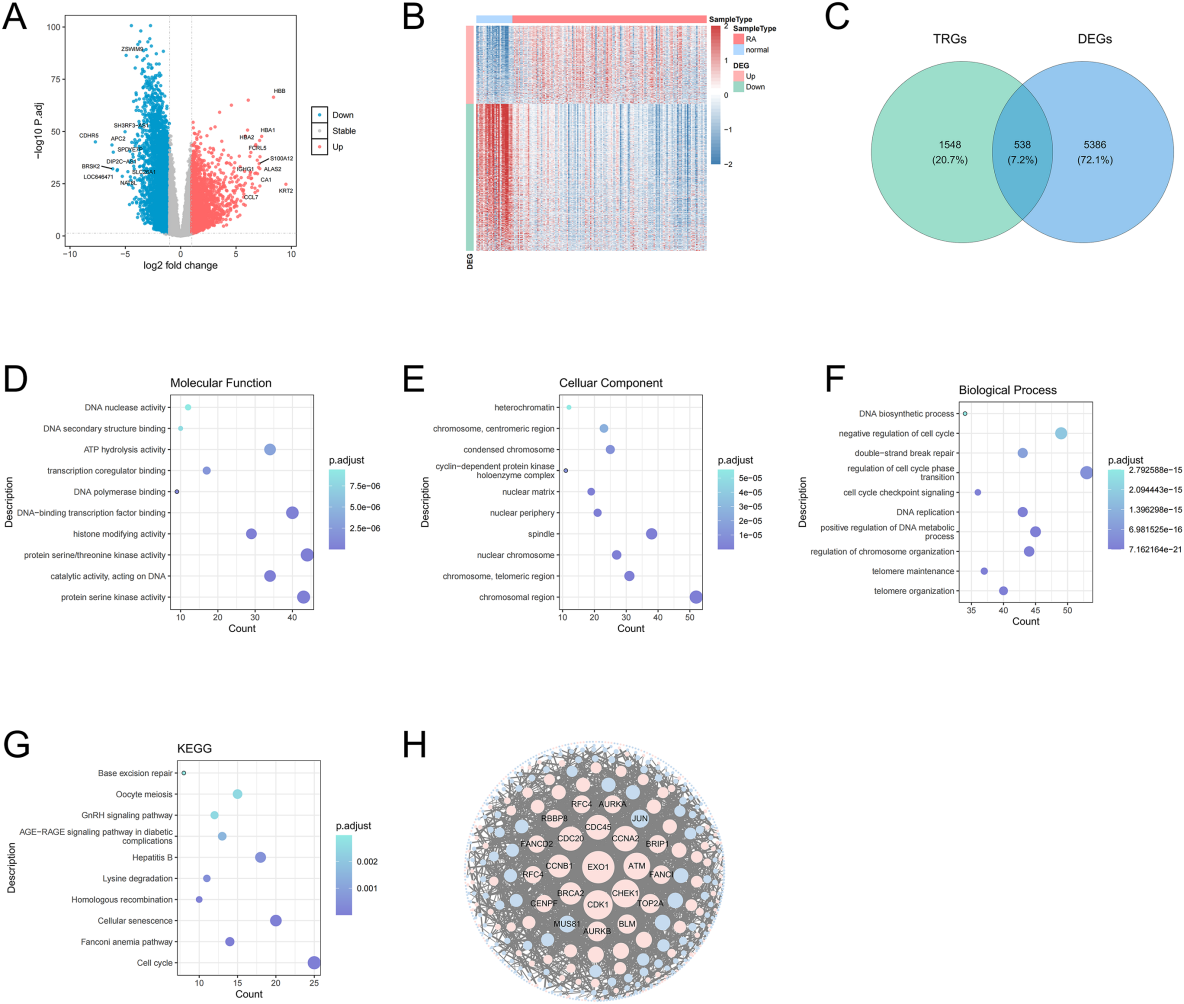
Volcano plot of DEGs between RA and control samples. Red dots represent upregulated genes and green dots represent downregulated genes **(A)**. Heat map of DEGs between RA and control samples **(B)**. Venn diagram of the intersection of DEGs and TRGs **(C)**. MF of candidate genes in GO terms, the size of the dots indicates the number of genes enriched with a larger dot indicating higher enrichment, and the color depth indicates the adj.*P* value, with darker color signifying a smaller adj.*P* value, indicating the more significant the enrichment **(D)**. Top 10 CC of DEGs in GO terms **(E)**. Top 10 BP of DEGs in GO terms **(F)**. Top 10 pathways from the KEGG pathways analysis **(G)**. The PPI network of candidate genes indicated the interactions among the candidate genes **(H)**.

A total of 5,924 DEGs were obtained in RA, including 2,060 up-regulated genes and 3,864 down-regulated genes. The volcano plot labeled the first 10 differentially expressed up/down-regulated genes (**Figure 1A**). The heat plot displayed all differentially expressed up/down-regulated genes between RA and control groups (**Figure 1B**). The 538 candidate genes were obtained by the intersection of DEGs and TRGs (**Figure 1C**). GO analysis enriched 706 functions, including 560 BP, 43 CC, and 103 MF (**Supplementary Table 3**). In GO analysis, The MF was significantly enriched in protein serine kinase activity and catalytic activity acting on DNA (**Figure 1D**). The CC significantly enriched in chromosomal region, nuclear chromosome, and spindle (**Figure 1E**). the BP was significantly enriched in telomere organization, telomere maintenance, and DNA replication (**Figure 1F**). KEGG analysis enriched 280 pathways (**Supplementary Table 4**), mainly including cell cycle, lysine degradation, and base excision repair (**Figure 1G**). The PPI network demonstrated interactions between candidate genes (**Figure 1H**).

### 3.2 Identification of biomarkers

**Figure 2:**
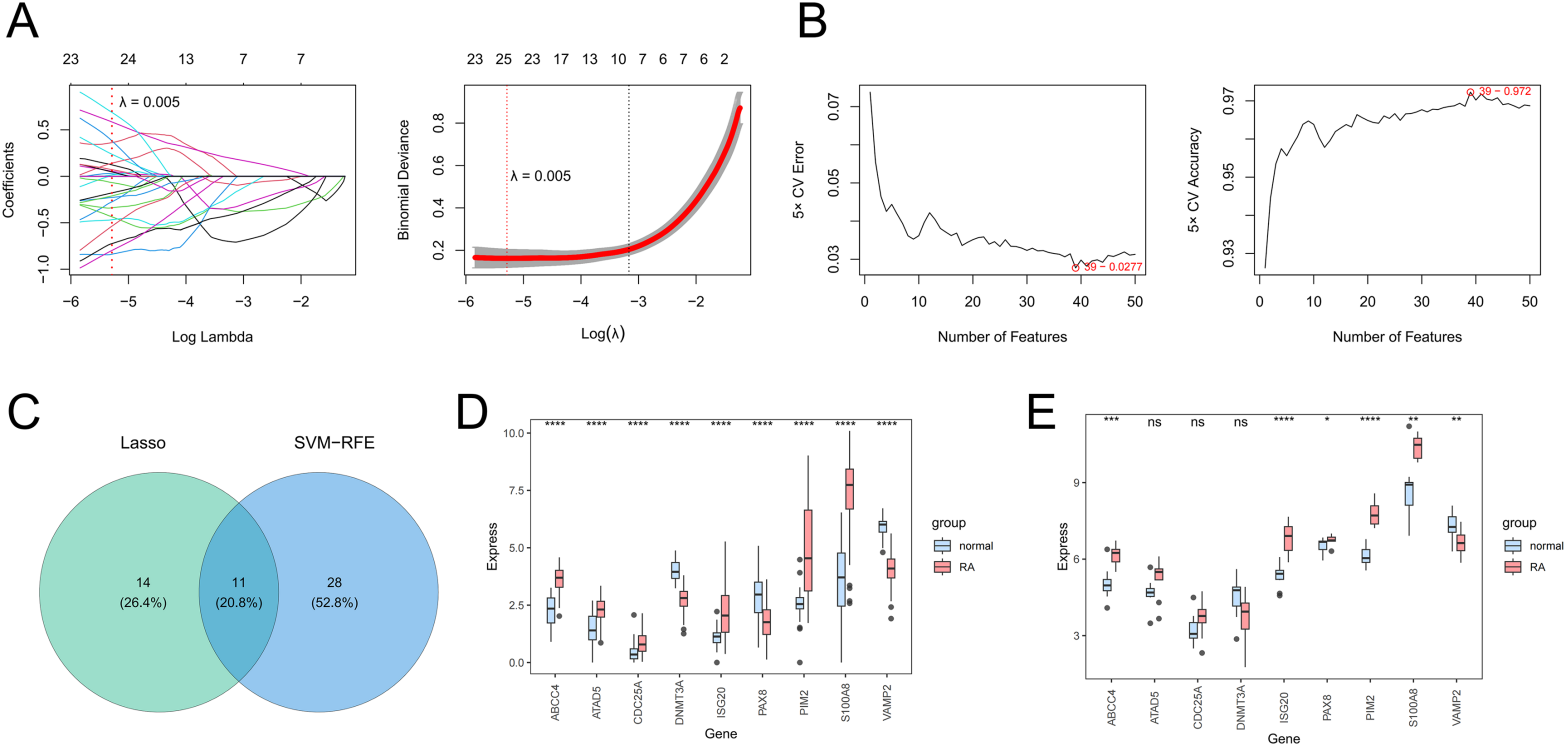
The screening of candidate genes by the LASSO algorithm **(A)** and the SVM-RFE algorithm **(B)**. The Venn diagram presents the core genes shared by LASSO and SVM-RFE **(C)**. The expression levels of core genes between RA and control samples in training **(D)** and validation **(E)** sets. *P < 0.05; **P < 0.01; ***P < 0.001; ****P < 0.0001; ns, no significance.

Next, the 25 genes were obtained by the LASSO algorithm (lambda. min = 0.005) (**Figure 2A**), and the 39 genes were obtained by the SVM-RFE algorithm (**Figure 2B**), so the 11 core genes were obtained by the intersection of the LASSO results and SVM-RFE results (**Figure 2C**). Whether GSE89408 or GSE55235, the AUC values of 9 core genes were greater than 0, so 9 core genes were regarded as candidate biomarkers (**Supplementary Figure 1**). In GSE89408, the expression levels of ABCC4, S100A8, PIM2, and ISG20 in RA samples were significantly higher than those in control samples, while the expression levels of VAMP2 in RA samples were significantly lower than those in control samples (**Figure 2D**). In GSE55235, the results were consistent with those of GSE89408, so ABCC4, S100A8, VAMP2, PIM2, and ISG20 were considered biomarkers for subsequent analysis (**Figure 2E**).

### 3.3 Nomogram model analysis for RA

**Figure 3:**
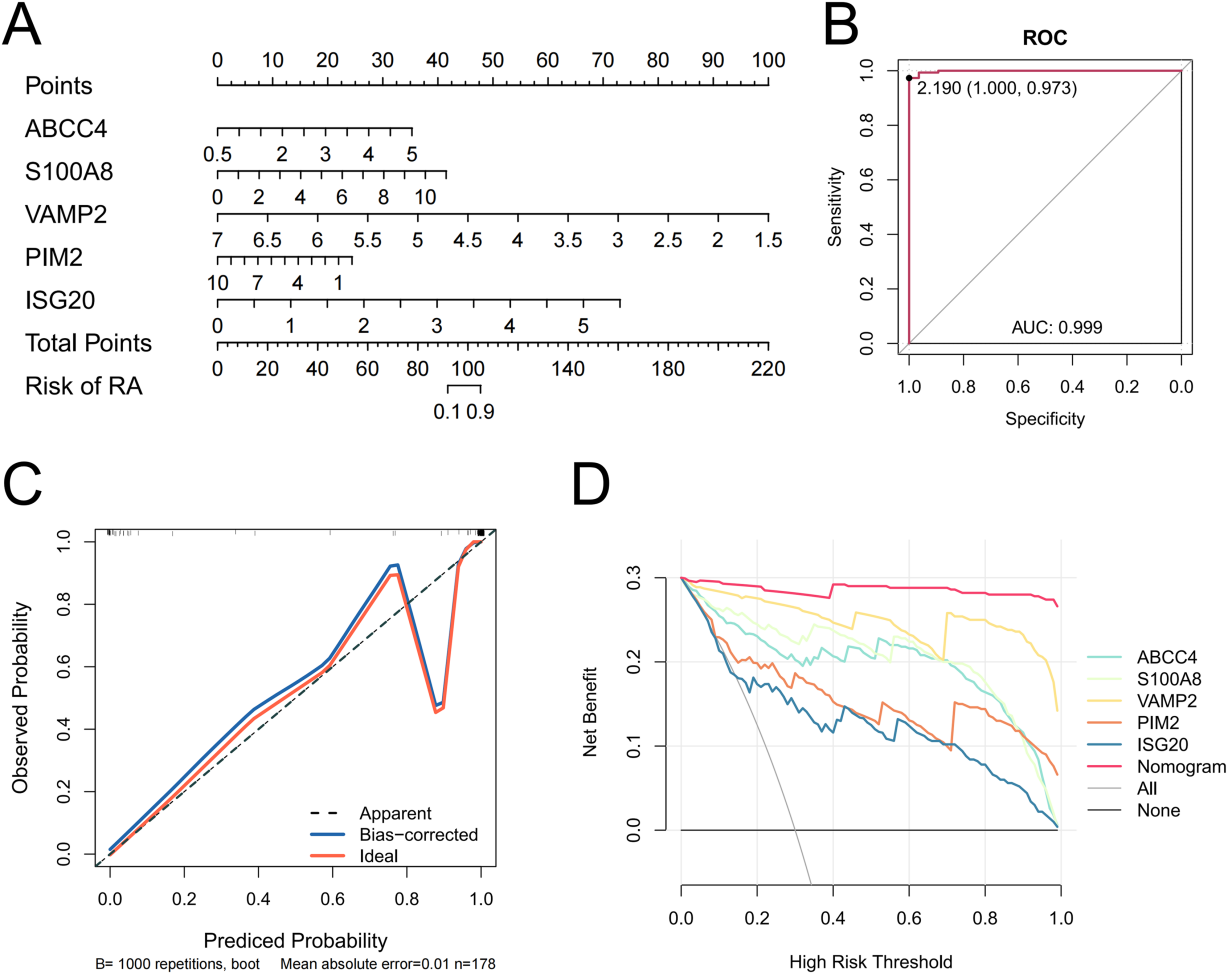
Nomogram of biomarkers **(A)**. A ROC curve of the nomogram **(B)**. Calibration curves evaluating the predictive ability of the nomogram **(C)**. DCA is evaluating the predictive ability of the biomarkers and the nomogram **(D)**.

The nomogram model constructed by biomarkers indicated biomarkers had a diagnostic ability for RA (**Figure 3A**). The AUC value was 0.999, so the model had an excellent ability to predict the incidence rate for RA (**Figure 3B**). The calibration curve coincided with the ideal curve, so the nomogram had excellent diagnostic ability (**Figure 3C**). The net benefit of the nomogram model was greater than those of any single biomarker, so the nomogram model had an excellent clinical effect (**Figure 3D**).

### 3.4 Enrichment pathway of biomarkers

**Figure 4:**
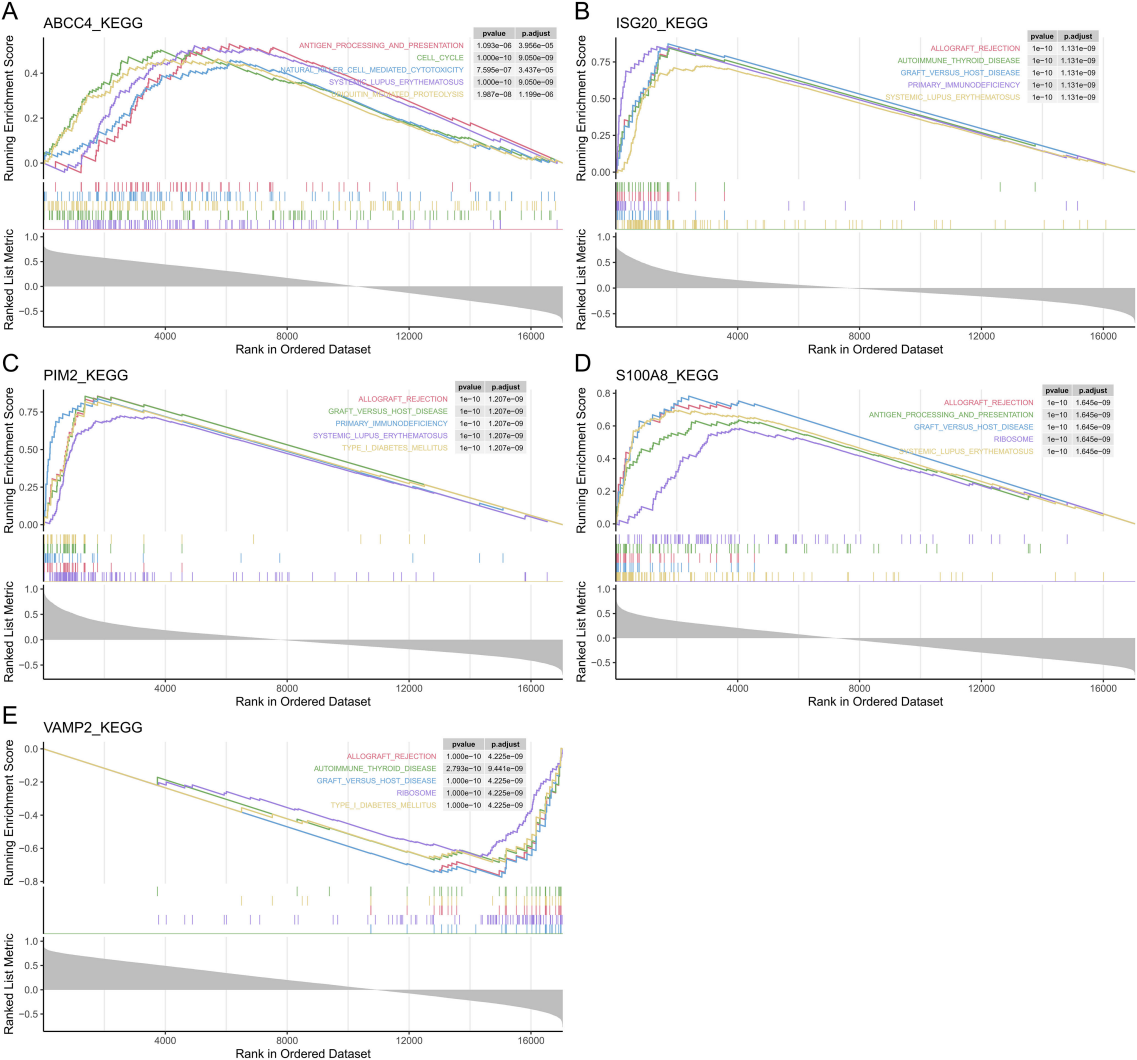
The GSEA of the biomarkers. The top 5 GSEA-enriched pathways of ABCC4 **(A)**, ISG20 **(B)**, PIM2 **(C)**, S100A8 **(D)**, and VAMP2 **(E)**.

According to GSEA, ABCC4 significantly enriched 38 pathways, including cell cycle, RNA degradation, and systemic lupus erythematosus (**Figure 4A**, **Supplementary Table 5**). ISG20 significantly enriched 63 pathways, including graft versus host disease, allograft rejection, and cell cycle (**Figure 4B**, **Supplementary Table 6**). PIM2 significantly enriched 58 pathways, including cell cycle, circadian rhythm mammal, and gap junction (**Figure 4C**, **Supplementary Table 7**). S100A8 significantly enriched 54 pathways, including systemic lupus erythematosus, cell cycle, and allograft rejection (**Figure 4D**, **Supplementary Table 8**). VAMP2 significantly enriched 33 pathways, including allograft rejection, type I diabetes mellitus, and protein export (**Figure 4E**, **Supplementary Table 9**). In conclusion, most of the biomarkers were enriched in allograft rejection and cell cycle.

### 3.5 Distribution of biomarkers in subcells and chromosomes

**Figure 5:**
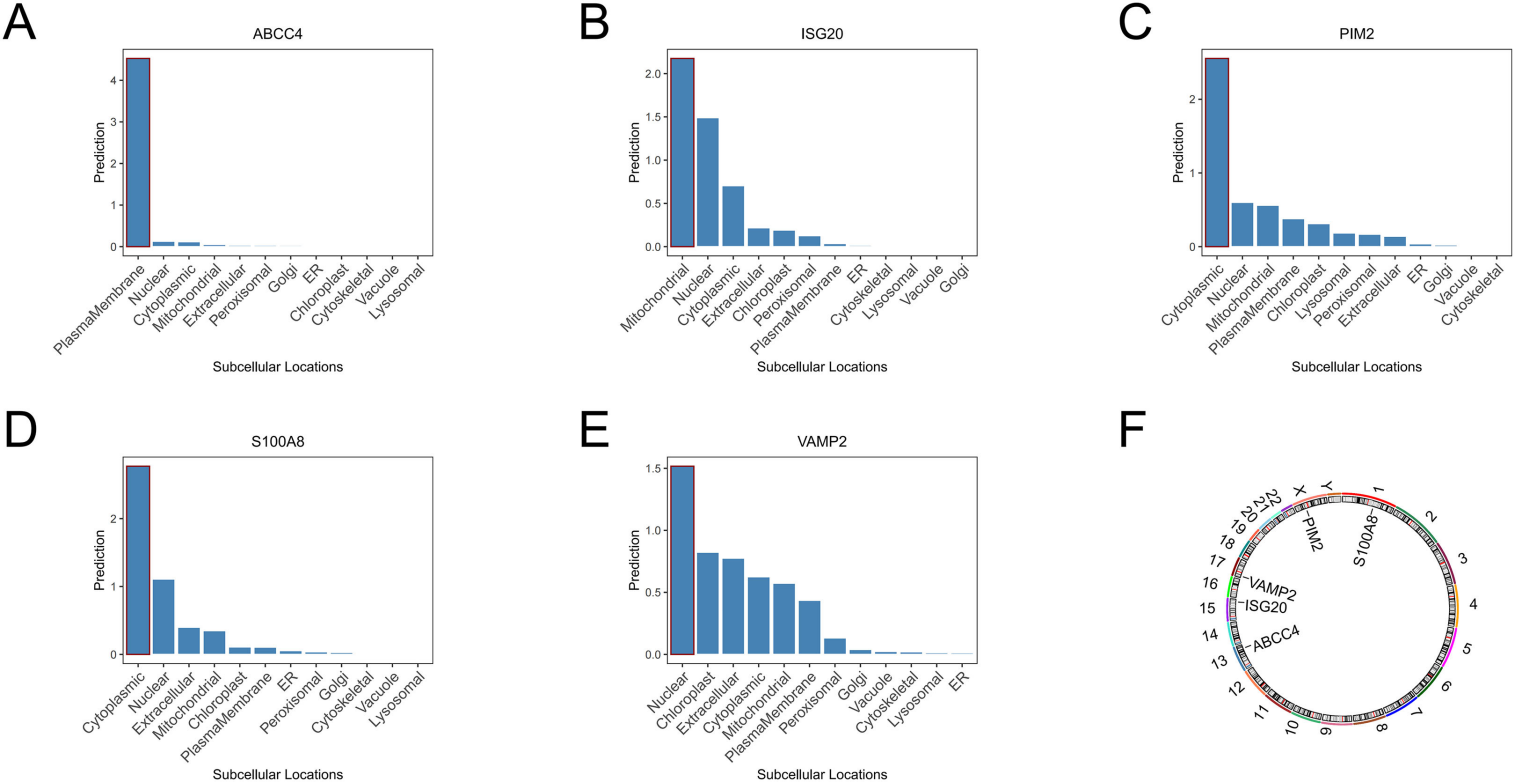
Subcellular localization analysis of ABCC4 **(A)**, ISG20 **(B)**, PIM2 **(C)**, S100A8 **(D)**, and VAMP2 **(E)**. Chromosomal localization analysis of biomarkers **(F)**.

According to subcellular localization analysis, ABCC4 was highly expressed on the plasma membrane, ISG20 was highly expressed in the mitochondria, PIM2 and S100A8 were highly expressed in the cytoplasm, and VAMP2 was highly expressed in the nucleus (**Figures 5A–E**). According to the distribution plot of biomarkers on chromosomes, S100A8 was distributed on chromosome 1, ABCC4 was distributed on chromosome 13, ISG20 was distributed on chromosome 15, VAMP2 was distributed on chromosome 17, and PIM2 was distributed on chromosome X (**Figure 5F**).

### 3.6 Immune cell analysis

**Figure 6:**
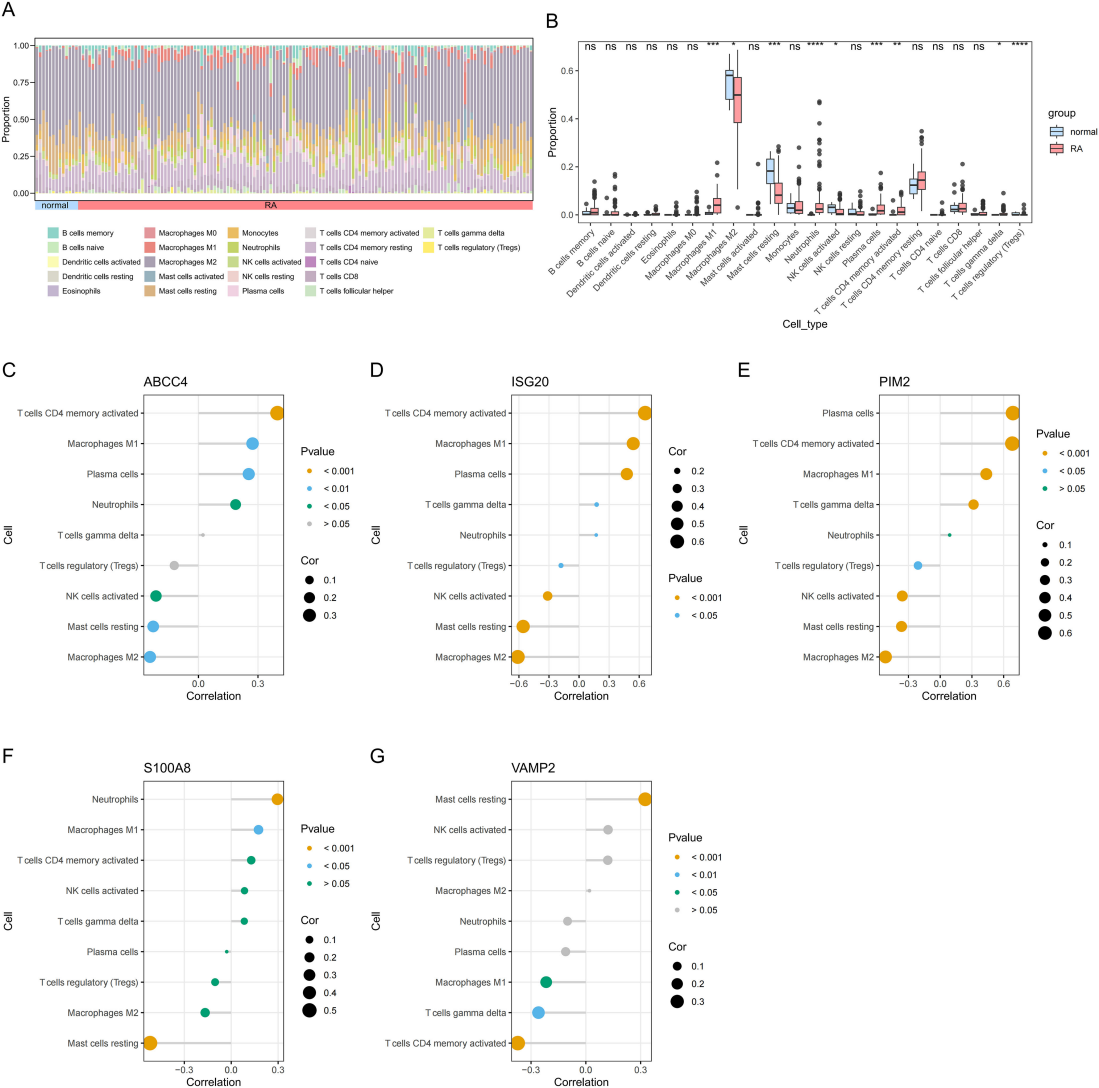
Immune cell abundance histogram of 22 immune cells in the RA and control samples **(A)**. Box plot of differences in immune cell infiltration between RA and control samples **(B)**. Correlation between ABCC4 **(C)**, ISG20 **(D)**, PIM2 **(E)**, S100A8 **(F)**, VAMP2 **(G)**, and differential immune cells in RA. *P < 0.05; **P < 0.01; ***P < 0.001; ****P < 0.0001; ns, no significance.

The abundance of 22 types of immune cells in RA and control samples was displayed by the CIBERSORT algorithm (**Figure 6A**). There were significant differences in 9 immune cells between RA and control samples, including M1 macrophages, M2 macrophages, resting mast cells, neutrophils, activated natural killer (NK) cells, plasma cells, activated CD4 memory T cells, delta gamma T cells, and regulatory T cells (**Figure 6B**). ABCC4 was positively correlated with activated CD4 memory T cells (cor = 0.40, *P* < 0.001) and negatively correlated with M2 macrophages (cor = -0.24, *P* < 0.001) (**Figure 6C**, **Supplementary Table 9**). ISG20 was positively correlated with activated CD4 memory T cells (cor = 0.66, *P* < 0.001) and negatively correlated with M2 macrophages (cor = -0.61, *P* < 0.001) (**Figure 6D**, **Supplementary Table 10**). PIM2 was positively correlated with plasma cells (cor = 0.69, *P* < 0.001) and negatively correlated with M2 macrophages (cor = -0.52, *P* < 0.001) (**Figure 6E**, **Supplementary Table 10**). S100A8 was positively correlated with neutrophils (cor = 0.30, *P* < 0.001) and negatively correlated with resting mast cells (cor = -0.52, *P* < 0.001) (**Figure 6F**, **Supplementary Table 10**). VAMP2 was positively correlated with resting mast cells (cor = 0.33, *P* < 0.001) and negatively correlated with activated CD4 memory T cells (cor = -0.37, *P* < 0.001) (**Figure 6G**, **Supplementary Table 10**).

### 3.7 Functional analysis of biomarkers

**Figure 7:**
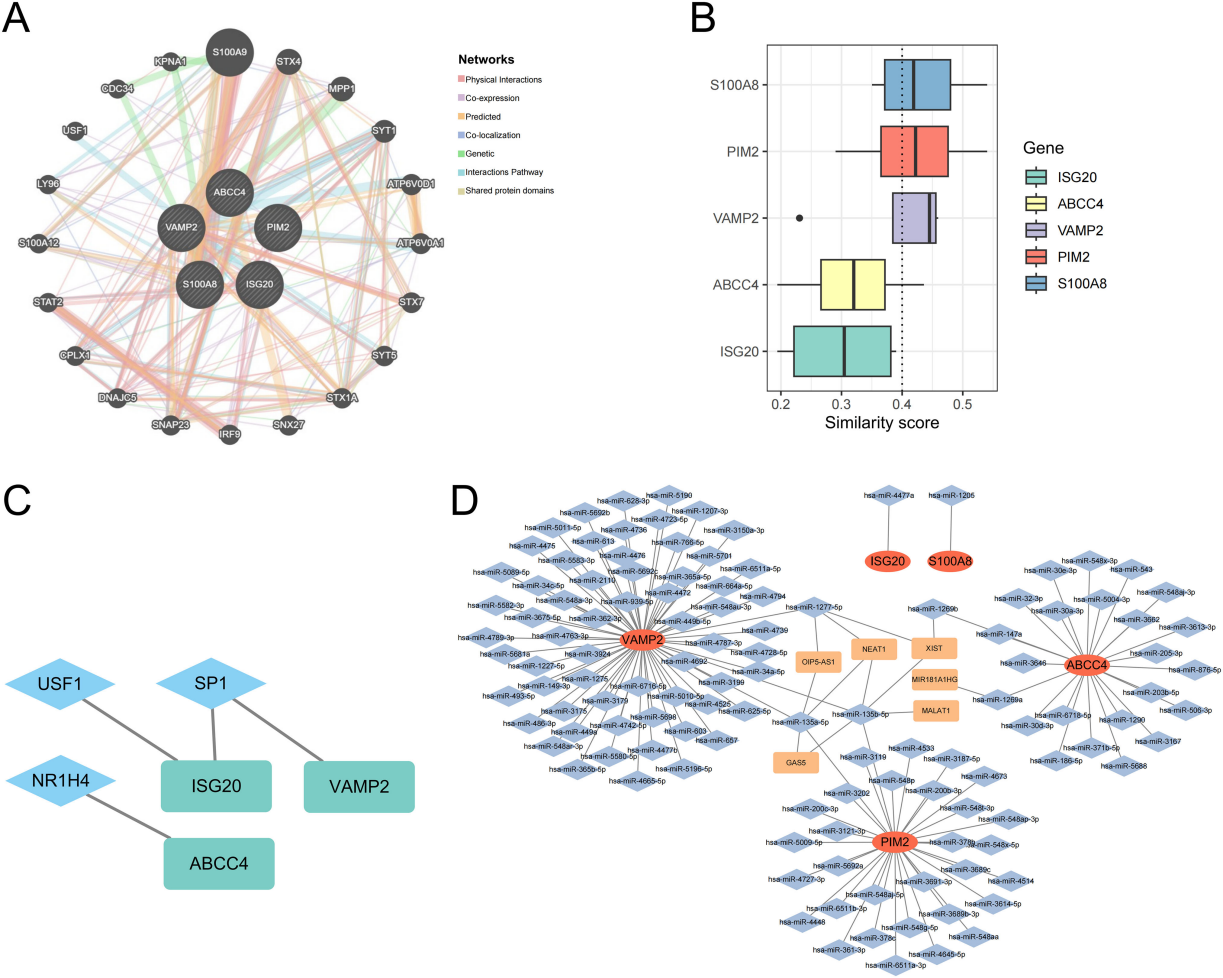
Co-expression networks of biomarkers **(A)**. Functional similarity analysis of biomarkers **(B)**. The molecular regulatory network between biomarkers and TFs **(C)**. mRNA-miRNA-lncRNA regulatory network of biomarkers **(D)**.

Co-expression networks of biomarkers and other genes were mainly involved in physical interactions, co-expression, predicted, co-localization, genetic, interactions pathways, and shared protein domains (**Figure 7A**). The functional similarity scores between S100A8, PIM2, VAMP2, and other biomarkers were greater than 0.4, indicating high functional similarity (**Figure 7B**). The 3 TFs were targeted biomarkers, including USF1, SP1, and NR1H4 (**Figure 7C**). The 121 miRNAs and 6 lncRNAs were obtained from the database; VAMP2 targeted more miRNAs and lncRNAs (**Figure 7D**).

### 3.8 Binding energy analysis of biomarkers and drugs

**Figure 8:**
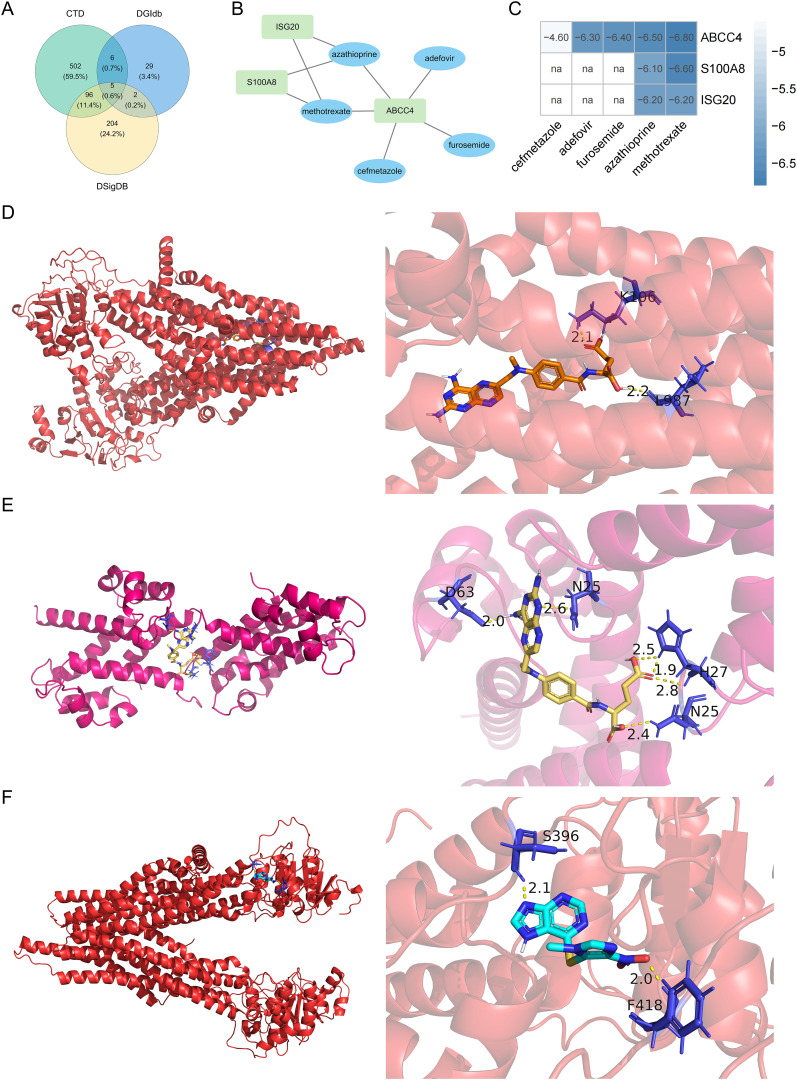
Venn diagram of the intersection of the drugs in the DGIdb, CTD, and DSigDB databases **(A)**. Biomarkers-drug interaction network **(B)**. Binding energy of molecular docking between drugs and biomarkers **(C)**. Molecular docking between ABCC4 and methotrexate **(D)**, molecular docking between S100A8 and methotrexate **(E)**, and molecular docking between ABCC4 and azathioprine **(F)**. na, not available.

The 5 drugs were predicted by the database, including methotrexate, adefovir, furosemide, azathioprine, and cefmetazole (**Figure 8A**). ABCC4 interacted with all drugs (**Figure 8B**). ABCC4 had the strongest binding energy with methotrexate (**Figure 8C**). The binding energy of ABCC4 and methotrexate was -6.80 kcal/mol, the binding energy of S100A8 and methotrexate was -6.60 kcal/mol, and the binding energy of ABCC4 and azathioprine was -6.50 kcal/mol (**Figures 8D–F**).

### 3.9 RT-qPCR results

**Figure 9:**
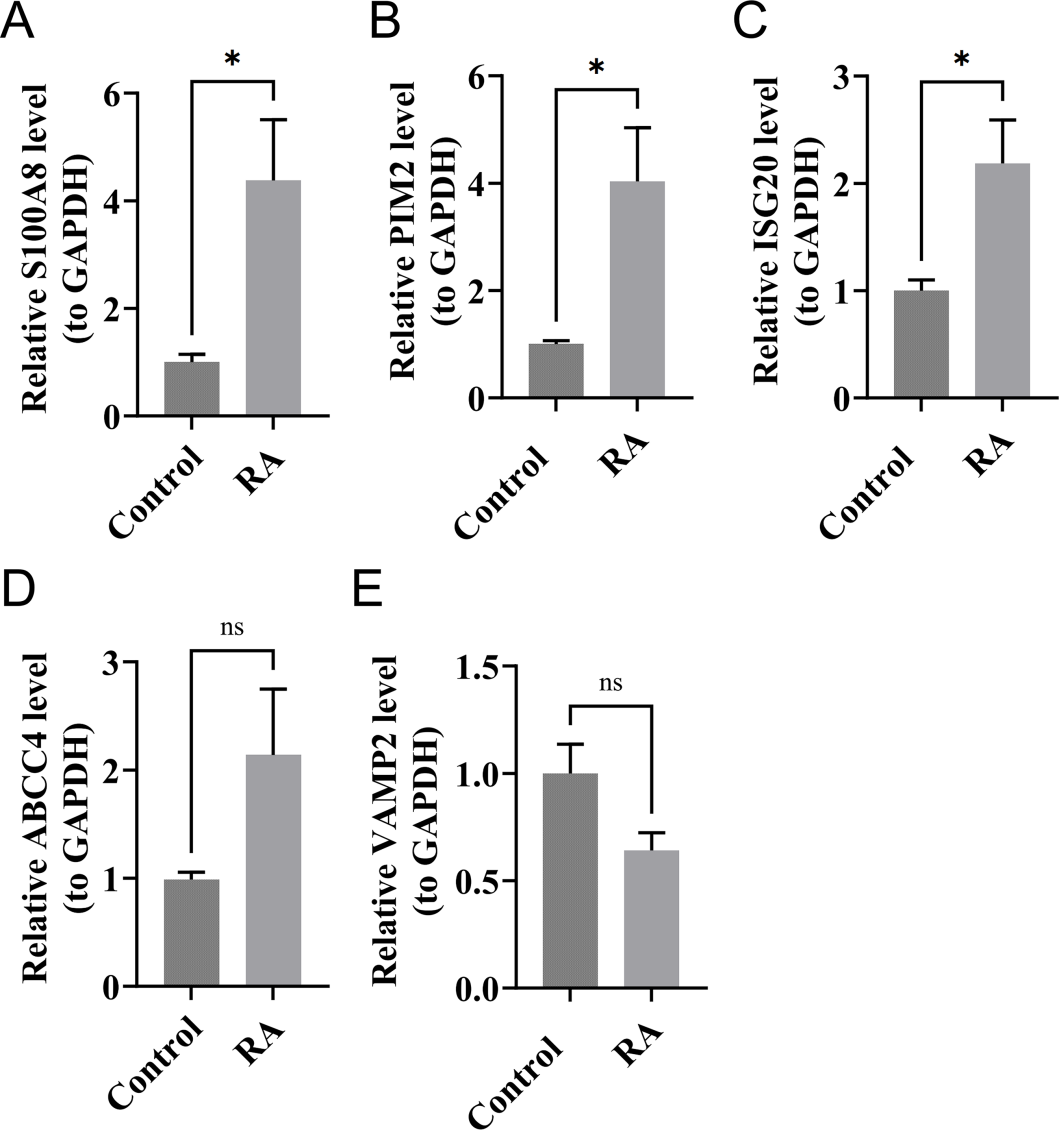
The expression levels of S100A8 **(A)**, PIM2 **(B)**, ISG20 **(C)**, ABCC4 **(D)**, and VAMP2 **(E)** in RA and control samples by RT-qPCR. *P < 0.05; ns, no significance.

The expression levels of S100A8, PIM2, and ISG20 in RA samples were significantly higher than those in the control samples (*P* < 0.05). Although the expression level of ABCC4 in RA samples was higher than that in the control samples, the difference was not significant. Similarly, the expression of VAMP2 in RA samples was lower than that in the control samples, but the result was also not significant (**Figure 9**). Compared with the bioinformatics analysis results, the expression of biomarkers in *in vitro* experiments was consistent with the results, which increased the reliability of bioinformatics analysis results.

## 4 Discussion

RA is a chronic inflammatory disease with multiple comorbidities, characterized by synovial hyperplasia, often leading to irreversible joint erosion and disability (37). Telomere length shortening has been observed in various diseases, including RA. Studies have reported premature telomere shortening in lymphocytes and hematopoietic stem cells of RA patients compared to healthy controls, which may be associated with immunosenescence (38). Other research has detected shorter telomere lengths and increased oxidative stress in peripheral blood cells of RA patients (39). However, the relationship between telomeres and the development of RA is not fully understood. Studying telomeres may lead to new insights into the pathophysiology of RA and provide ideas for RA treatment.

In this study, we conducted differential gene analysis on transcriptome data from RA patients and control samples, identifying a total of 5,924 DEGs, including 2,060 upregulated and 3,864 downregulated genes. By intersecting the DEGs with TRGs, we obtained 538 candidate genes. Through PPI network analysis, GO enrichment analysis, and KEGG pathway enrichment analysis, we deeply explored the biological functions and related pathways of these candidate genes. Subsequently, using machine learning methods, we selected 11 core genes related to RA and finally identified five biomarkers (ABCC4, S100A8, VAMP2, PIM2, and ISG20) through expression verification and ROC analysis. The nomogram constructed based on these biomarkers showed high accuracy and clinical benefit in predicting RA. GSEA revealed that most biomarkers were enriched in pathways related to allograft rejection and the cell cycle. Additionally, transcription factor regulatory network and molecular regulatory network analyses were conducted to understand the regulatory relationships among biomarkers. Subcellular and chromosomal localization analyses revealed that ABCC4 was localized to the plasma membrane, ISG20 to mitochondria, PIM2 and S100A8 to the cytoplasm, and VAMP2 to the nucleus. Immune infiltration analysis identified nine differentially abundant immune cell types, with most biomarkers significantly correlated with CD4 memory T cells. Functional analyses utilizing GeneMANIA, Friends, and molecular regulatory networks provided further insights into the roles of the biomarkers. Finally, drug prediction identified five drugs interacting with the biomarkers. Binding energy analysis revealed strong binding affinity between ABCC4, S100A8, and ISG20 with methotrexate. The results of the *in vitro* experiment were consistent with those of the bioinformatics analyses, thereby validating the reliability of the bioinformatics analysis results. Compared to the control samples, the expression levels of S100A8, PIM2, and ISG20 were significantly elevated in RA samples, while no significant differences were observed in the expression levels of ABCC4 and VAMP2, which may be attributed to inter-sample heterogeneity.

S100A8, also known as a subunit of calprotectin, is a member of the S100 protein family and is expressed in the cytoplasm and nucleus of various cells, predominantly localized in the cytoplasm (40). S100A8 is involved in regulating cell cycle progression and differentiation, and it plays a key role in innate immune activation. Elevated levels of calprotectin have been observed in the synovial fluid, plasma, and serum of patients with RA (41). Recent studies indicate that calprotectin can serve as a biomarker for RA, correlating more effectively with active inflammatory disease than traditional acute phase reactants (42). S100A8 proteins were significantly elevated during persistent inflammation, suggesting their potential as biomarkers of disease activity. Inhibition of S100A8 has been shown to ameliorate severe inflammation (43), indicating that the S100A8 protein may be a viable therapeutic target for RA patients. In addition, S100A8 was found to promote oxidative stress by regulating neutrophil chemotaxis and activation, leading to telomere damage in hepatocytes, which accelerated the progression of liver disease and the formation of hepatocellular carcinoma (44). Therefore, we hypothesize that the mechanism of action between S100A8 and telomere damage may similarly affect the onset and progression of RA.

ISG20 was an RNA exonuclease that belongs to the yeast RNA exonuclease 4 homolog subfamily. It may affect telomere repair and maintenance by regulating RNA degradation and the stability of gene expression. Through its exonuclease activity, ISG20 can degrade viral RNA, thereby inhibiting viral proliferation (45). It plays a significant role in the antiviral innate immune response of host cells (46). In addition, studies have shown that the expression of ISG20 in synovial macrophages of RA patients is significantly higher than that in the control group (47), which is consistent with the findings of this study. Therefore, ISG20 is expected to serve as a potential biomarker and therapeutic target for RA.

ABCC4, an ATP-binding cassette (ABC) transporter, is primarily responsible for ATP binding and hydrolysis, as well as substrate recognition and transport (48). It plays a crucial role in maintaining intracellular and extracellular balances of drugs and chemicals, participating in multidrug resistance and detoxification processes, and is capable of transporting organic anions and other molecules (49). Inhibition of ABCC4 suppresses the extracellular transport of cAMP and enhances intracellular PKA activity, downstream gene expression, and glucocorticoid-induced anti-inflammatory responses (50). Hence, future research efforts can be focused on identifying drug targets capable of inhibiting ABCC4 expression, aiming to enhance the efficacy of anti-inflammatory drugs in the treatment of RA.

VAMP2, also known as synaptobrevin2, is the prototypical v-SNARE. The v-SNARE consists of a family of proteins known as VAMPs, which were located on the surface of SVs at nerve terminals. Notable homologous VAMP isoforms include VAMP1, VAMP2, VAMP3, VAMP4, VAMP7, and VAMP8 (51). VAMP2 has a well-characterized and conserved role in synaptic function, primarily involved in the assembly of effective SNARE complexes, Ca^2+^-dependent exocytosis of synaptic vesicles, and rapid endocytosis at hippocampal synapses (51). VAMP2 is a core component of the SNARE complex. Strong immunoreactivity of VAMP2 has been observed in vesicular glutamate transporter-immunoreactive reticulated nerve endings (52). Although there is currently no direct evidence linking VAMP2 to RA, considering that RA patients often suffer from neuropathy and pain symptoms, the immunoreactivity of VAMP2 at nerve endings and its critical role in synaptic vesicle trafficking suggest a possible association with the neuropathological mechanisms of RA. Specifically, VAMP2 may indirectly contribute to pain perception and inflammatory responses in RA by influencing signal transduction and immune reactions at nerve endings. Therefore, exploring the specific mechanism of VAMP2’s role in RA neuropathy and investigating its potential as a therapeutic target is of great significance.

PIM2, a proto-oncogene belonging to the serine/threonine kinase family, was implicated in various intracellular signaling processes that influence cell growth and division, promoting cell survival and proliferation (53). This function potentially contributes to preventing premature telomere damage, thereby playing a crucial role in maintaining telomere stability. PIM2 also affects the expression of IL-6, a cytokine central to the pathogenesis of RA. The overexpression of PIM2 has been shown to enhance IL-1β- and TNF-α-induced IL-6 expression (54). In summary, these genes were directly or indirectly related to inflammatory responses, cell proliferation, and immune regulation in RA; thus, they were promising as biomarkers and therapeutic targets for RA.

Most biomarkers were found to be enriched in the contexts of allograft rejection and the cell cycle. Kimura et al. were the first to propose the significant role of allograft inflammatory factor-1 (AIF-1) in the development and progression of RA (55). The expression of AIF-1 was significantly elevated in infiltrating mononuclear cells and synovial fibroblasts from RA patients (56). AIF-1 induced the proliferation of cultured synovial cells, and the production of IL-6 by synovial fibroblasts and blood monocytes increased following AIF-1 stimulation (55). Research indicates that AIF-1, functioning as a cytokine, demonstrates early and sustained expression in chronically rejected allogeneic heart grafts while remaining unexpressed in both the heart graft and the host heart. AIF-1 may serve as a predictor for allograft rejection and is associated with a lower risk of rejection (57, 58). It is widely accepted that the overproliferation and decreased apoptosis of fibroblast-like synoviocytes can contribute to synovial hyperplasia. Studies have shown that inhibiting G1/S cell cycle progression can suppress the proliferation of fibroblast-like synoviocytes in adjuvant-induced arthritis (59, 60). This suggests that biomarkers, by modulating pathways such as allograft rejection and the cell cycle, may provide new paradigms for the treatment of RA. Therefore, further research is warranted to elucidate the specific mechanisms through which biomarkers regulate RA via pathways involving allograft rejection and the cell cycle.

Multiple immune cells infiltrate the local joint microenvironment, collaborating to promote the progression of RA. The results of immune infiltration identified nine differentially expressed immune cell types between RA patients and control subjects. Correlation analysis revealed that most biomarkers were significantly associated with activated CD4 memory T cells. T cells were key pathogenic drivers of RA autoantibody production and were essential for synoviocyte proliferation, neoangiogenesis, and the erosion of cartilage and bone (61). Memory CD4 T cells were particularly important in the context of autoimmune diseases due to their long-lived nature, effective response to antigens, and unique capacity to mediate recurrent autoimmune responses (62). Genetic risk loci associated with RA were preferentially mapped to enhancers and promoters that were active in CD4 T cell subsets (63). Aberrant activation of memory CD4 T cells plays a crucial role in the initiation and perpetuation of RA. Activated memory CD4 T cells migrating between the bloodstream and synovium express cytokines and activation markers that contribute to tissue damage in RA (64). Therefore, exploring the changes in the immune microenvironment of RA, especially the abnormal activation and infiltration of memory CD4 T cells, is of great significance for untangling the pathophysiology of RA, identifying new therapeutic targets, and developing treatment strategies based on immunomodulation.

Methotrexate interacts with biomarkers and exhibits the strongest binding energy. It inhibits several key enzymes involved in the synthesis pathways of folate, methionine, adenosine, and *de novo* nucleotides. As an antifolate drug, it possesses antiproliferative and anti-inflammatory effects (47). Methotrexate has been established as an effective and fast-acting disease-modifying antirheumatic drug, widely utilized in the treatment of RA. S100A8, ABCC4, and ISG20 had strong drug interactions with methotrexate. S100A8 is secreted locally by phagocytes at inflammatory sites, where it exerts numerous autocrine and paracrine pro-inflammatory effects on both phagocytes and endothelium. S100A8, a calcium-binding protein expressed by neutrophils, is a component of calprotectin. It may play an important role in assessing the therapeutic efficacy of methotrexate and disease activity, and it is more closely related to the therapeutic response to methotrexate (65). Furthermore, methotrexate, which targets S100A8, when combined with fluid resuscitation, significantly reduces the transcription level of S100A8 and inflammatory cytokine content in blood, organ damage, and mortality in severely burned mice (66). ABCC4, recognized as the first mammalian nucleotide exporter among ABC transporters, confers cellular resistance to nucleoside analog antiretroviral drugs (48). ABC transporters play a crucial role in the efflux of methotrexate from cells, with approximately 80-90% of the administered methotrexate dose being excreted through urine within 48 hours, primarily via the ABCC4 transporter during the first 8–12 hours (67). Although there is currently no direct research available on the relationship between ISG20 and methotrexate, future studies can further explore this connection, providing more insights for the treatment of RA. Azathioprine, a prodrug of 6-mercaptopurine, is utilized for the treatment of inflammatory diseases such as RA, systemic lupus erythematosus, and inflammatory bowel disease. Research indicates that in the management of patients with severe ANCA-associated vasculitis, azathioprine exerts an immunosuppressive effect during induction of remission, thereby lowering S100A8 and subsequently reducing the risk of recurrence for patients (68). Therefore, we believe that these biomarkers have the potential to become new targets for RA treatment, providing new directions for the development of more effective RA therapeutics.

This study successfully identified biomarkers of TRGs associated with RA through transcriptome data analysis and machine learning algorithms and constructed a predictive model based on these biomarkers. These biomarkers exhibited excellent performance in the diagnosis of RA and were closely related to key pathways such as allograft rejection and cell cycle. Moreover, molecular docking experiments demonstrated strong binding affinities between ABCC4, S100A8, ISG20, and methotrexate, as well as other drugs, suggesting that these biomarkers could be potential targets for the treatment of RA.

However, there were limitations in this study. For instance, the research findings heavily rely on data quality, algorithm accuracy, and assumptions made during analysis, which may lead to data noise, false positives, and false negatives. Additionally, due to the complexity of the algorithms, the interpretability of the study may be somewhat limited. Given the complexity and diversity of biological systems, this study may not have fully untangled the actual biological processes. In bioinformatics analyses, ABCC4 interacted with methotrexate, adefovir, furosemide, azathioprine, and cefmetazole, suggesting its potential as a new therapeutic target for RA. However, in RT-qPCR experiments, although the expression level of ABCC4 in RA samples was higher than that in control samples, the difference was not significant. This may be due to the high heterogeneity of RA patients, coupled with the small sample size in this study, which did not cover different subgroups of RA patients, resulting in the masking of gene expression differences. Therefore, future research should further explore the specific roles of these biomarkers in the pathophysiology of RA. The diagnostic and prognostic value of the target can be verified through cellular experiments, animal models, and techniques such as gene knockout and overexpression. Its potential as a therapeutic target can be further evaluated in larger clinical trials. Moreover, an in-depth investigation into the relationship between telomere length and key phenotypes of RA will provide crucial insights for the development of novel therapeutic strategies. Observing changes in telomerase activity through the *in vitro* cultivation of fibroblast-like synoviocytes from RA patients may offer further support for research in this field.

## Data Availability

The original contributions presented in the study are included in the article/**Supplementary Material**. Further inquiries can be directed to the corresponding author/s.

## Supplementary material

Supplementary Figure 1 ROC curves of the candidate biomarkers in GSE89408 **(A)** and GSE55235 **(B)**.
