# Supplementary figures and images for: Transcriptomic insights into the mechanism of action of telomere-related biomarkers in rheumatoid arthritis
Source: Front Immunol. 2025 May 22;16:1585895. doi: 10.3389/fimmu.2025.1585895 (PMC12137363; doi:10.3389/fimmu.2025.1585895)

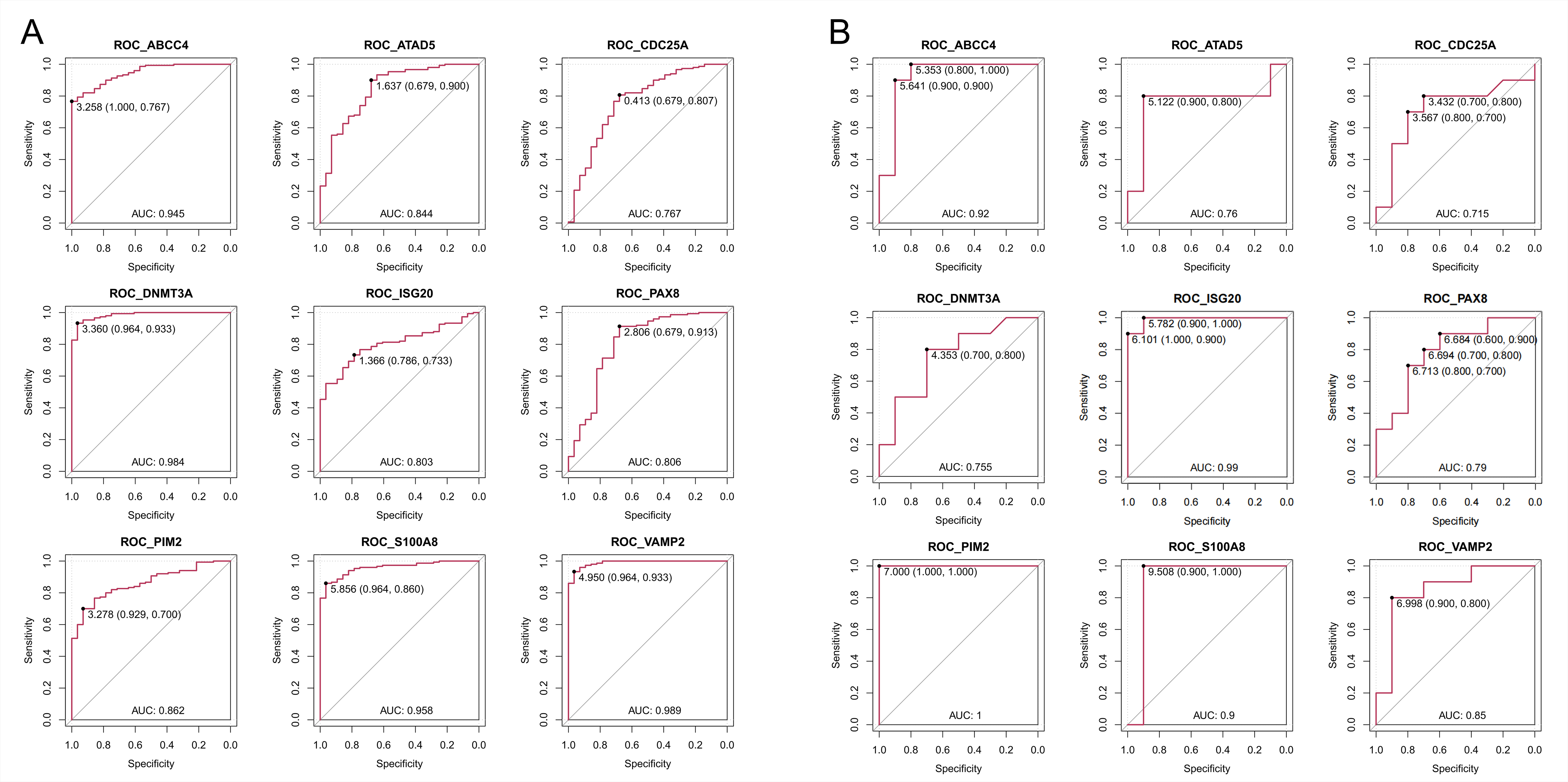

Supplement: Supplementary Figure 1 — ROC curves of the candidate biomarkers in GSE89408 (A) and GSE55235 (B). [file Image1.tif]
